# Identification of key immune-related genes in dilated cardiomyopathy using bioinformatics analysis

**DOI:** 10.1038/s41598-022-26277-w

**Published:** 2023-02-01

**Authors:** Feng Li, Tong-Yue Du, Li-Da Wu, Lei Zhang, Huan-Huan Liu, Zhen-Ye Zhang, Jie Zhang, Zhi-Yuan Zhang, Ling-Ling Qian, Ru-Xing Wang, Jian-Feng Hao

**Affiliations:** 1 Department of Cardiology, Wuxi People’s Hospital Affiliated to Nanjing Medical University, No. 299, Qingyang Road, Wuxi, 214023 China; 2 Department of Critical Care Medicine, The Second Hospital of Nanjing, Nanjing University of Chinese Medicine, Nanjing, 210003 China; 3 Wuxi School of Medicine, Jiangnan University, Wuxi, 214122 China; 4 Department of Cardiopulmonary Rehabilitation, Wuxi Tongren Rehabilitation Hospital Affiliated to Nanjing Medical University, Wuxi, 214122 China

**Keywords:** Cardiology, Cardiac device therapy, Cardiovascular biology

## Abstract

Dilated cardiomyopathy (DCM) is characterized by the left ventricular dilatation and impaired myocardial systolic dysfunction with high mortality and morbidity. However, the underlying mechanisms remain elusive. We first identified the differentially expressed genes (DEGs) between the DCM and control group using two expression profiles from GSE3585 and GSE84796. Enrichment analysis was conducted to explore the potential mechanisms underlying DCM. A total of four algorithms, including key module of MCODE, degree, maximum neighborhood component (MNC), and maximal clique centrality (MCC), were used to identify the hub genes within Cytoscape. The correlation between hub genes and infiltrated immune cells was evaluated to determine potential immune-related genes. The expression analysis and diagnosis value analysis of potential immune-related genes were performed. Finally, the expression analysis with GSE57338 and relationship analysis with the comparative toxicogenomics database (CTD) were performed to identify the key immune-related genes in DCM. A total of 80 DEGs were screened for DCM. Enrichment analysis revealed that DEGs were involved in the immune-related pathological process. Immune infiltration analysis indicated a potentially abnormal immune response in DCM. Four up-regulated genes (C*OL1A2*, *COL3A1*, *CD53*, and *POSTN*) were identified as potential immune-related genes. Finally, three genes (C*OL1A2*, *COL3A1*, and *POSTN*) were determined as the key immune-related genes in DCM via expression analysis with a validation set (GSE57338) and relationship analysis with CTD. Our study suggested that the upregulated C*OL1A2*, *COL3A1*, and *POSTN* might be the key immune-related genes for DCM. Further studies are needed to validate the underlying mechanisms.

## Introduction

Dilated cardiomyopathy (DCM), one of the severe cardiac disorders, is characterized by the left ventricle dilatation and systolic function impairment, with an estimated prevalence ranged from 0.2 to 0.4% in the U.S. adult population^1^. A plenty number of studies have revealed multiple risk factors and diseases, such as family history of cardiomyopathy, myocarditis, and toxic effects medications, may be implicated in the DCM. Moreover, though several treatment strategies for DCM are recommended, including drug therapy, cardiac resynchronization therapy, cardiac cell therapy, and heart transplantation, challenges remain, such as irreversible disease progression, unsatisfied therapy effect, and relatively poor prognosis (only 30–40% survival rates of 5- to 10-year)^2^.

It has been reported that multiple physiopathology mechanisms were implicated in the development and progression of DCM, such as oxidative stress, abnormal immune response, inflammation, fibrosis and genetic changes^3^. However, precise molecular mechanisms underlying the occurrence of DCM, especially the abnormal immune response, remain unclear. Importantly, increasing evidence has indicated that the abnormality of immune responses may play a significant role in the initiation and progression of DCM. An RNA-sequencing study for classified DCM phenotype showed a total of three distinct molecular profiles underlying DCM, in which auto-immune phenogroup could significantly connect with the activation of TNF-signaling and NFκB-signaling, leading to myocardial injury in the progression of DCM^4^. A recent study based on the blood specimens of DCM patients suggested the changes of circulating immune cells level (e.g., T cells) may be involved in the immunomodulatory function in the development of DCM, which was expected to be a potential novel therapeutic target^5^. Therefore, it has central significance to the identification of key immune-related genes and mechanisms in DCM.

In this study, we performed differentially expressed genes (DEGs) analysis between DCM patients and non-DCM patients using two DCM gene expression profiles from GSE3585 and GSE84796. Enrichment analysis was performed to explore the potential mechanisms underlying DCM. Then, a total of four algorithms were used within Cytoscape to identify the hub genes. Correlation analysis between hub genes and infiltrated immune cells was conducted to identify potential immune-related genes. The expression analysis and diagnosis value analysis of potential immune-related genes were performed. Finally, the expression analysis with another gene set (GSE57338) and relationship analysis with CTD were performed to identify the key immune-related genes in DCM.

## Methods

### Microarray data

**Figure 1:**
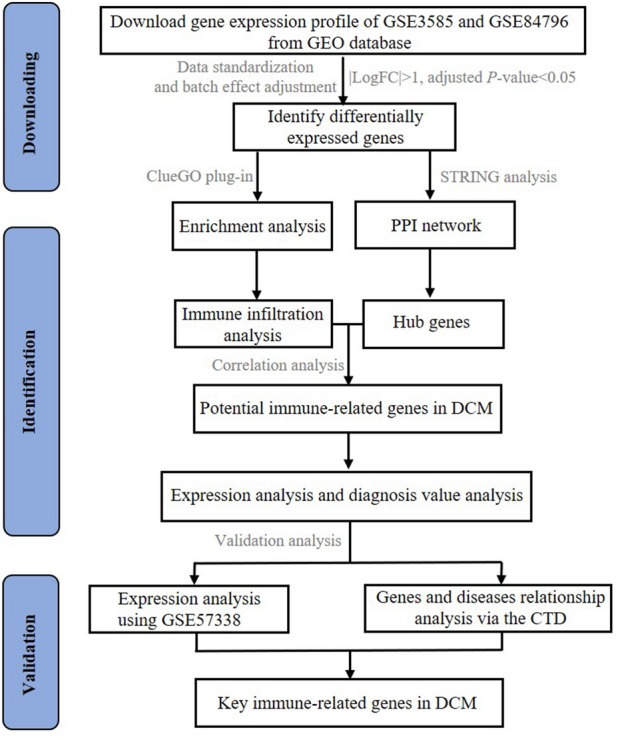
Flowchart of data analysis in this study. GEO: Gene Expression Omnibus; PPI: protein–protein interaction; DCM: dilated cardiomyopathy; CTD: comparative toxicogenomics database.

A total of three DCM gene expression profiles of GSE3585, GSE84796 and GSE57338 are acquired from NCBI Gene Expression Omnibus (GEO) (https://www.ncbi.nlm.nih.gov/geo/), which is a public and freely available database (Fig. 1). The first two DCM gene expression profiles were used as integrated set. The data set GSE3585, performed by GPL96 platform (Affymetrix Human Genome U133A Array), was collected from left ventricular tissue biopsy samples from 7 DCM patients and 5 non-DCM patients. The data set GSE84796, based on the platform GPL14550 (Agilent-028004 SurePrint G3 Human GE 8 × 60K Microarray), included 10 patients with severe chagas chronic cardiomyopathy (severe DCM) and 7 healthy patients for heart donation. The data set GSE57338, based on GPL11532 (Affymetrix Human Gene 1.1 ST Array) was used as a validation set for expression level of hub genes, including a total of 82 DCM patients and 136 non-DCM patients (Table 1).

**Table1:** The characteristic baseline of DCM gene expression profiles.

Author (year)	Series accession	Platform	Sample (DCM vs. non-DCM)	Application
Barth (2006)	GSE3585	GPL96	7 vs. 5	Integrated set
Laugier (2017)	GSE84796	GPL14550	10 vs.7	Integrated set
Liu (2015)	GSE57338	GPL11532	82 vs. 136	Validation set

DCM: dilated cardiomyopathy.

### Data standardization and batch effect adjustment

For data standardization, log_2_-transformation, background correction, and quantile normalization were performed on the raw gene expression profiles using the linear models for the microarray data (limma) algorithm. Then, based on the annotation file, the probe numbers were converted into gene symbols. In order to extract the common gene symbols from two datasets (GSE3585 and GSE84796), we used the removeBatchEffect function of the limma package (Version 3.52.2) in the R software to remove batch effects. The result of the data preprocessing was assessed by boxplot. The principal component analysis (PCA) plot was drawn to illustrate the samples before and after batch effect.

### Screening of differentially expressed differentially expressed genes (DEGs)

Fold change (FC), a widely useful threshold to identify the possible differentially expressed genes, is a filter for comparing the absolute expression value change between two groups. In this study, we evaluated the gene expression profile and screened the DEGs between DCM patients and non-DCM patients according to log_2_FC expression using the limma method. To avoid eliminating excessive potential immune-related genes in expression profiles, a relatively low cut-off value of adjust *P*-value < 0.05 and |log_2_FC| > 1) was set, and the heat maps of DEGs were presented by the R packages of “ggplot2” (version: 3.3.5). Moreover, the volcano plot related to DEGs was presented using the “ggplot2” packages (version: 4.1.2).

### Enrichment analysis of the DEGs

Gene Ontology (GO) knowledgebase is the world’s largest source of information on the functions of genes, and GO analysis consisted of multiple sections, including biological processes (BP), cellular components (CC), molecular functions (MF), and immune system process (ISP). Kyoto Encyclopedia of Genes and Genomes (KEGG) is a useful database resource for understanding high-level functions and utilities of the biological system from molecular-level information. Two significant parts of enrichment analysis, GO analysis and KEGG pathway analysis, of DEGs were performed using ClueGO (version 2.5.8). The ClueGO, a useful Cytoscape plug-in, could create and visualize the non-redundant biological terms for large clusters of genes in a functionally grouped network. In our study, *P*-value < 0.05 was set to show the terms and pathways with the medium network connectivity (kappa score of 0.4). Go terms tree interval shown the minimal level with three and the maximal level with five. The leading group terms were displayed based on the highest significance.

### Identification immune infiltration with DCM

CIBERSORT is a useful analytical tool to impute gene expression profiles and provide an estimation of the abundance of member cell types in a mixed cell population using gene expression data. In our study, CIBERSORT algorithm was performed to identify the immune infiltration between DCM patients and non-DCM patients. Leukocyte signature matrix (LM22), including gene expression matrix and source data, was analyzed to define a total of twenty-two immune cell subsets (https://cibersort.stanford.edu/), and generate the immune cell fractions matrix between DCM patients and non-DCM patients. The visualization of the results as a heatmap and box plot using the ‘Complexheatmap’ (version: 2.8.0) and ‘ggpubr’ (version: 0.4.0) package in R, respectively.

### Construction of the PPI network and screening the hub genes

The STRING is a database with the known and predicted protein–protein interactions, including physical (direct) and functional (indirect) associations, to facilitate understand the diseases-related mechanisms. In this study, PPI network was constructed via the STRING online analysis software (http://string-db.org/). According to the STRING online analysis results, the plug-in MCODE and CytoHubba in Cytoscape (version 3.7.1) were used to screen the hub genes. In plug-in MCODE, several parameters were set, including network scoring degree cutoff = 2, node score cutoff = 0.2, k-core = 2, and maximum depth = 100, to screen the closely connected regions and identify the key module in PPI network. In plug-in CytoHubba, a total of three algorithms, including gene connection degree, maximum neighborhood component (MNC), and maximal clique centrality (MCC) were applied and the top ten genes were screened, respectively. The hub genes were identified by overlapping the key module of MCODE and top 10 genes of three CytoHubba algorithms, which were presented via the Upset plot (version: 1.4.0) by R.

### Screening of potential immune-related genes in DCM

In our study, the relationship between hub genes and infiltrated immune cells was analyzed with the Spearman correlation analysis using R software for screening the potential immune-related genes in DCM. The results were visualized using the ‘ggpubr’ package. The potential immune-related genes in DCM were defined when a significant correlation was shown between hub genes and infiltrated immune cells.

### The expression and diagnosis value of the potential immune-related genes

The expression analysis of the potential immune-related genes between DCM patients and non-DCM patients was performed using Student’s t-test from GSE3585 and GSE84796. Meanwhile, the diagnosis value of the potential immune-related genes for DCM was assessed via receiver operating characteristic curve (ROC). A good discrimination was defined that the area under ROC curve (AUC) was more than 0.7. The visualization of the results was achieved by GraphPad Prism software (version 7.00).

### Identification of the key immune-related genes in DCM

For identification of the key immune-related genes in DCM, the expression analysis with validation set (GSE57338) and relationship analysis with CTD were performed. The comparative toxicogenomics database (CTD) is a publicly available database, which mainly provides manually curated information about chemical–disease relationships, gene–disease relationships, and chemical–gene or protein interactions (http://ctdbase.org/). In this study, relationship analysis of the key immune-related genes with cardiovascular diseases (including cardiovascular disease, cardiomyopathies, heart disease, and ventricular dysfunction) and immune-related diseases (including immune system disease and autoimmune disease) was performed using CTD (data updated on March 2, 2022), in which inference scores represented the level of association between disease and genes.

## Results

### Data procession

The result of data standardization for the two DCM gene expression profiles was shown in Supplementary Fig. 1A. PCA plot showed a complete intersection between gene expression profiles after batch removal (Supplementary Fig. 1B,C), indicating the two DCM gene expression profiles could serve as a batch of data for subsequent analysis.

### Identification of DEGs between DCM patients and non-DCM patients

**Figure 2:**
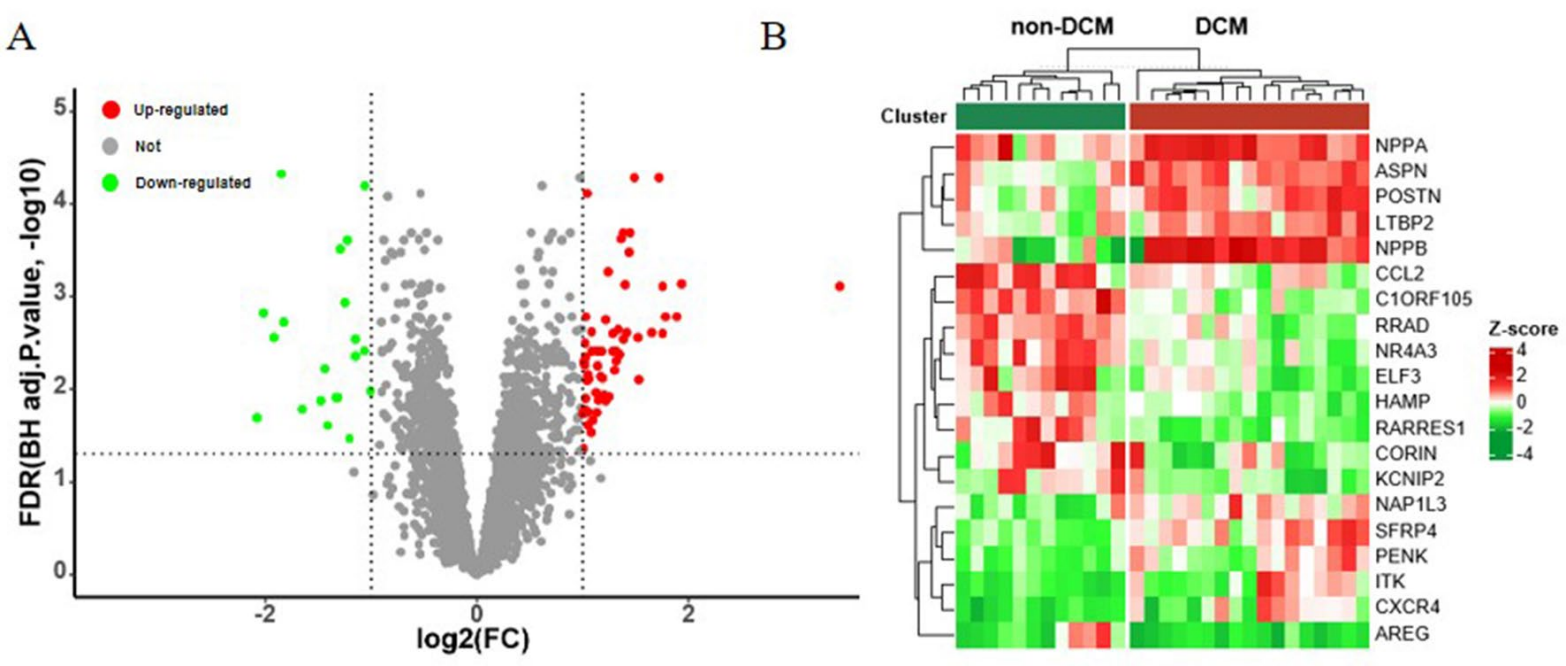
Identification of DEGs in DCM samples. (**A**) The expression volcano plot of the differential gene expression between DCM and non-DCM samples; (**B**) The expression heatmap of the top ten up-regulated and down-regulated DEGs between DCM and non-DCM samples. DEGs: differentially expressed genes; DCM: dilated cardiomyopathy.

After data procession on the dataset GSE3585 and GSE84796, we identified a total of 80 DEGs between DCM patients and non-DCM patients, including 60 up-regulated and 20 down-regulated genes. The volcano plot for all genes was illustrated in Fig. 2A. The expression heatmap for top ten DEGs of up- and down-regulated genes was shown in Fig. 2B.

### Enrichment analysis of the DEGs

**Figure 3:**
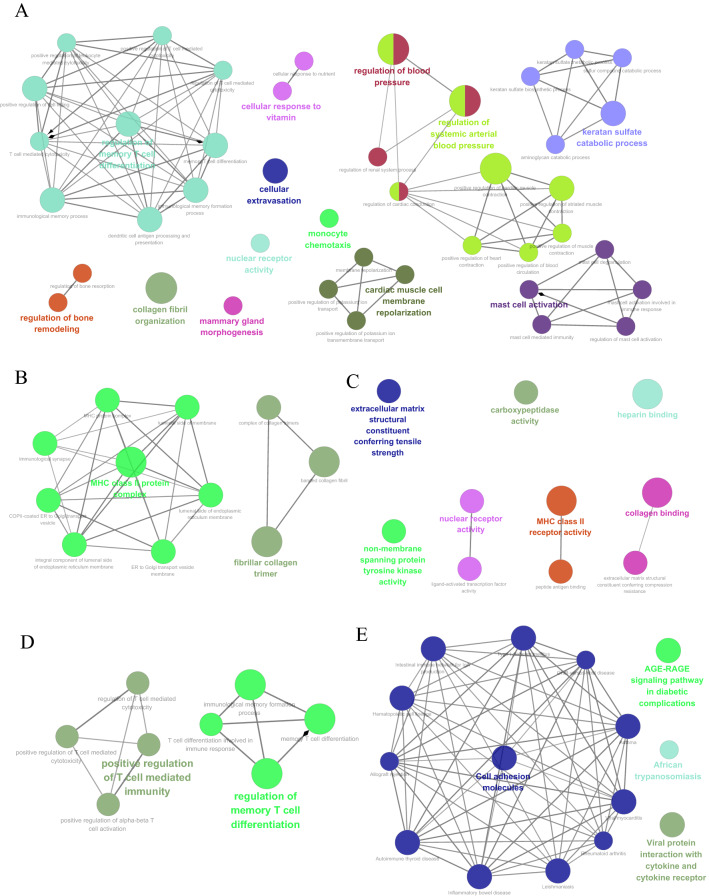
Enrichment analysis for DEGs using ClueGO. (**A**) Biological processes related enrichment; (**B**) cellular component related enrichment; (**C**) molecular function related enrichment; (**D**) immune system processes related enrichment; (**E**) KEGG enrichment. DEGs: differentially expressed genes; KEGG: Kyoto Encyclopedia of Genes and Genomes. *P*-value < 0.05 was set to show the terms and pathways with the medium network connectivity (kappa score of 0.4). The leading group terms were displayed based on the highest significance. Each group is presented by the label with the most significant term or pathway, which was circled by the dotted circle. The node size represents the term or pathway enrichment significance.

GO analysis was performed on the DEGs between DCM patients and non-DCM patients. The enriched biological processes were mainly related to regulation of memory T cell differentiation (GO: 0043380), cellular response to vitamin (GO: 0071295), cardiac muscle cell membrane repolarization (GO: 0099622), regulation of blood pressure (GO: 0008217), regulation of systemic arterial blood pressure (GO: 0003073), mast cell activation (GO: 0045576), and keratan sulfate metabolic process (GO: 0042339) (Fig. 3A). The cellular components were mainly involved in major histocompatibility complex (MHC) class II protein complex (GO: 0042613) and fibrillar collagen trimer (GO: 0005583) (Fig. 3B). Enriched molecular functions mainly included MHC class II receptor activity (GO: 0032395), nuclear receptor activity (GO: 0004879), and collagen binding (GO: 0005518) (Fig. 3C). Meanwhile, enriched immune system processes were mainly involved in the regulation of memory T cell differentiation (GO: 0043380) and positive regulation of T cell mediated immunity (GO: 0002711) (Fig. 3D). Figure 3E showed KEGG pathway analysis, and multiple immune-related pathways were enriched, including cell adhesion molecules (KEGG: 04514), viral protein interaction with cytokine and cytokine (KEGG: 04061), autoimmune thyroid disease (KEGG: 05320), and inflammatory bowel disease (KEGG: 05321).

### Immune infiltration analysis between DCM patients and non-DCM patients

**Figure 4:**
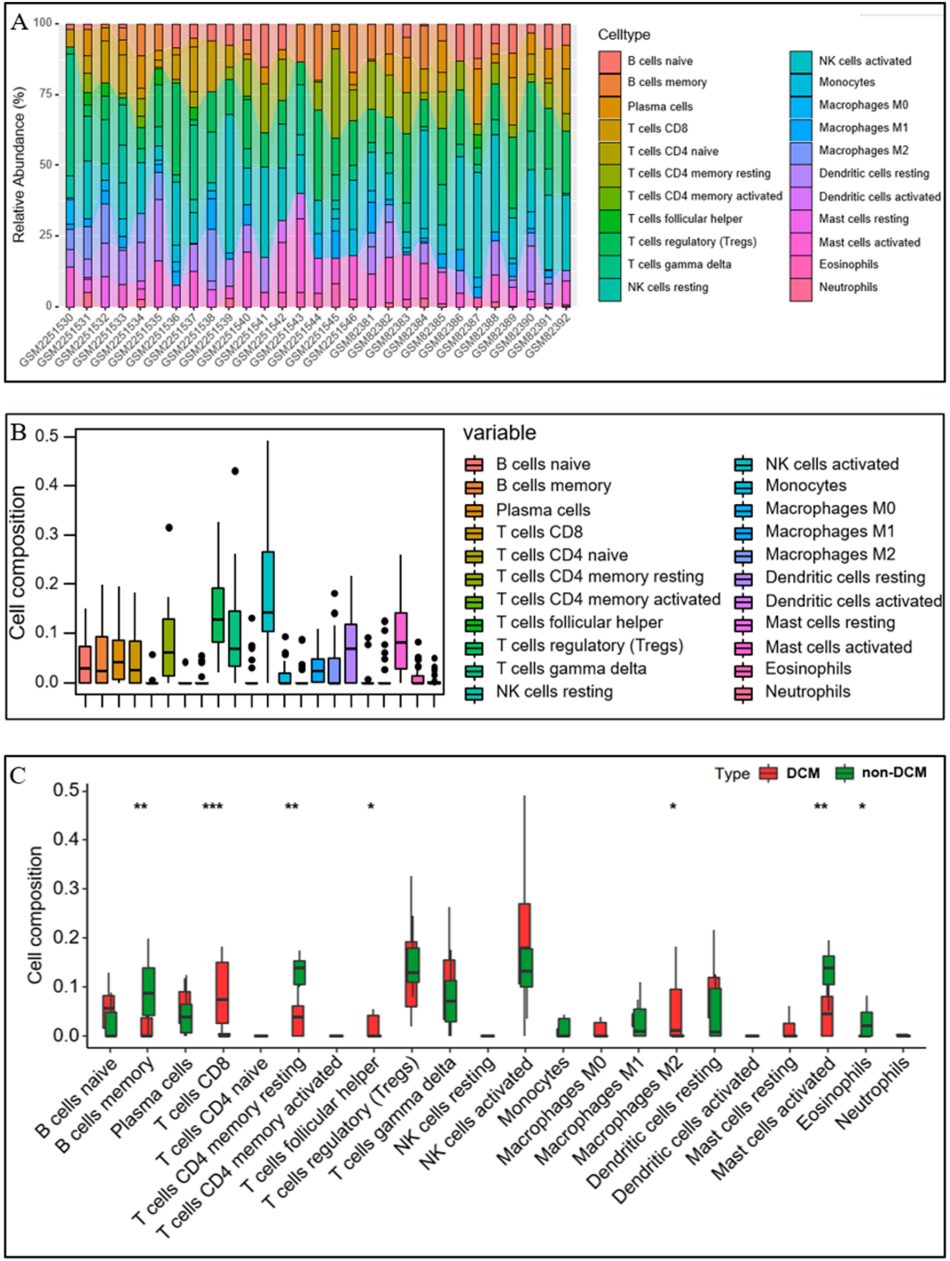
Immune infiltration analysis between DCM patients and non-DCM patients. (**A**) The percentage of twenty-two subpopulations of immune cells for each sample in DCM and non-DCM. (**B**) The percentage of twenty-two subpopulations of immune cells in total samples. (**C**) The difference of immune cells between DCM patients and non-DCM patients. DCM: dilated cardiomyopathy. *P < 0.05; **P < 0.01; ***P < 0.001.

CIBERSORT algorithm was performed to identify the immune infiltration between DCM patients and non-DCM patients. The proportion of twenty-two immune cell subsets between DCM patients and non-DCM patients was shown in Fig. 4A. We found that most major immune cell subpopulations, including activated NK cells, regulatory T cells, and activated mast cells, are presented in both DCM and non-DCM hearts (Fig. 4B). Moreover, compared to non-DCM hearts, DCM hearts showed a higher proportion of CD8(+) T cells, T follicular helper cells, and M2 macrophages, as well as a lower proportion of memory B cells, CD4(+) memory resting T cells, activated mast cells and eosinophils (Fig. 4C).

### Screening the hub genes in DCM

**Figure 5:**
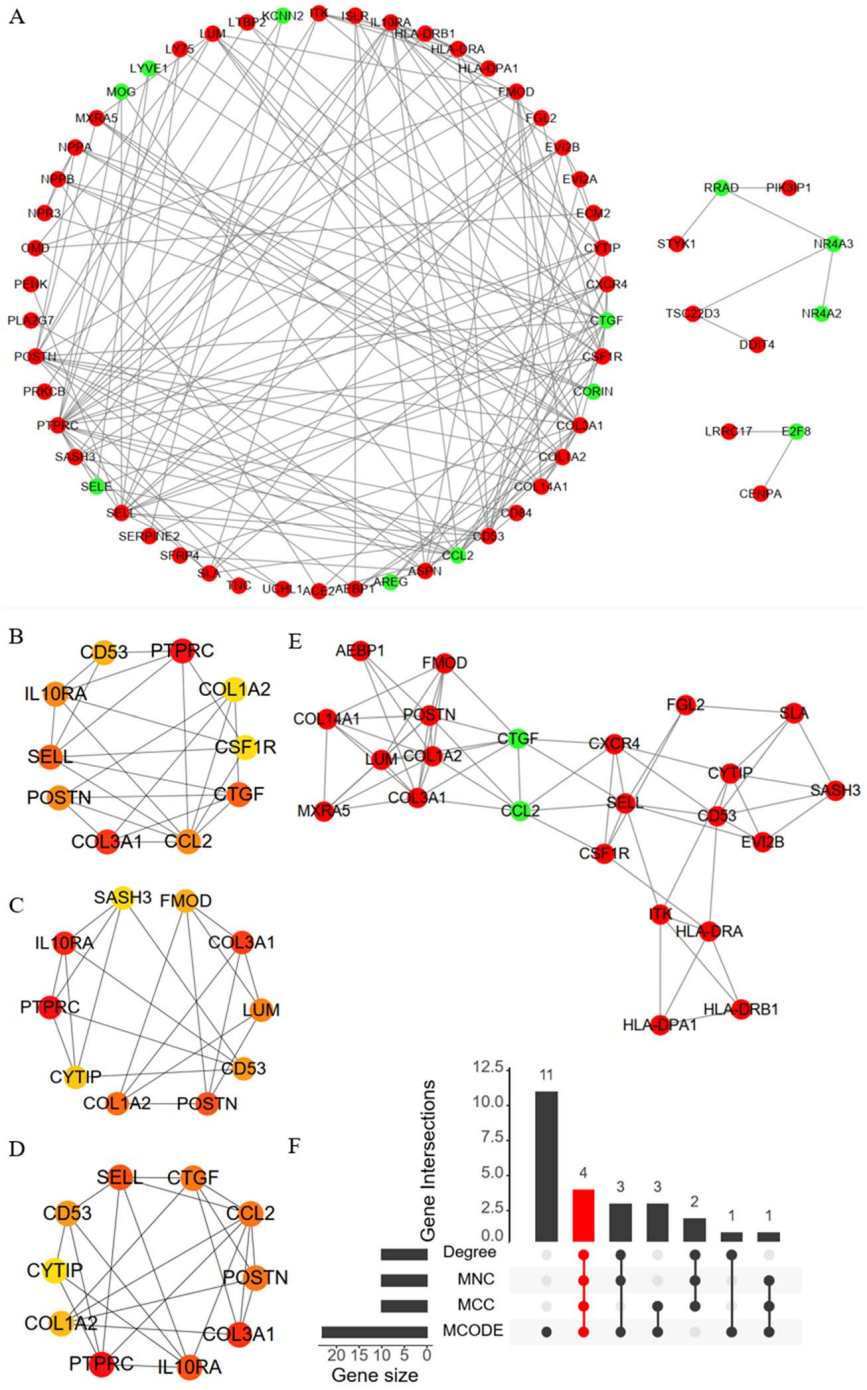
Construction of PPI network and identification of the hub genes in DCM. (**A**) PPI network of DEGs was constructed using STRING; A total of three algorithms from CytoHubba, including (**B**) Degree algorithm, (**C**) MCC algorithm, and (**D**) MNC algorithm, were used for the top ten genes; (**E**) The key module within the PPI network was identified from MCODE; (**F**) The hub genes were identified via the Upset plot. PPI: protein–protein interaction; DEGs: differentially expressed genes; MCC: maximal clique centrality; MNC: maximum neighborhood component. Red color represents up-regulated genes and green color represents down-regulated genes in (**A**) and (**E**); Advanced ranking is represented by a redder color in (**B**–**D**).

IN this study, we constructed PPI network based on the STRING database to further explore the potential relationship among proteins encoded by key genes, and the results showed a total of 60 nodes and 161 edges in the PPI network (Fig. 5A). Then, to comprehensive and accurate screen the hub DEGs, two plug-ins, including a total of three algorithms, were used within Cytoscape. In plug-in CytoHubba, four algorithms, including gene connection degree, MNC, and MCC were performed and the top ten genes were shown in Fig. 5B–D, respectively. In plug-in MCODE, the key module within the PPI network was identified and shown in Fig. 5E, including a total of 23 nodes and 66 edges. Importantly, for identifying the hub genes, the key module of MCODE and top ten genes with three algorithms of CytoHubba were overlapped, and finally a total of four hub genes were identified, including C*OL1A2*, *COL3A1*, *CD53*, and *POSTN* (Fig. 5F).

### Correlation analysis of hub genes and infiltrated immune cells

**Figure 6:**
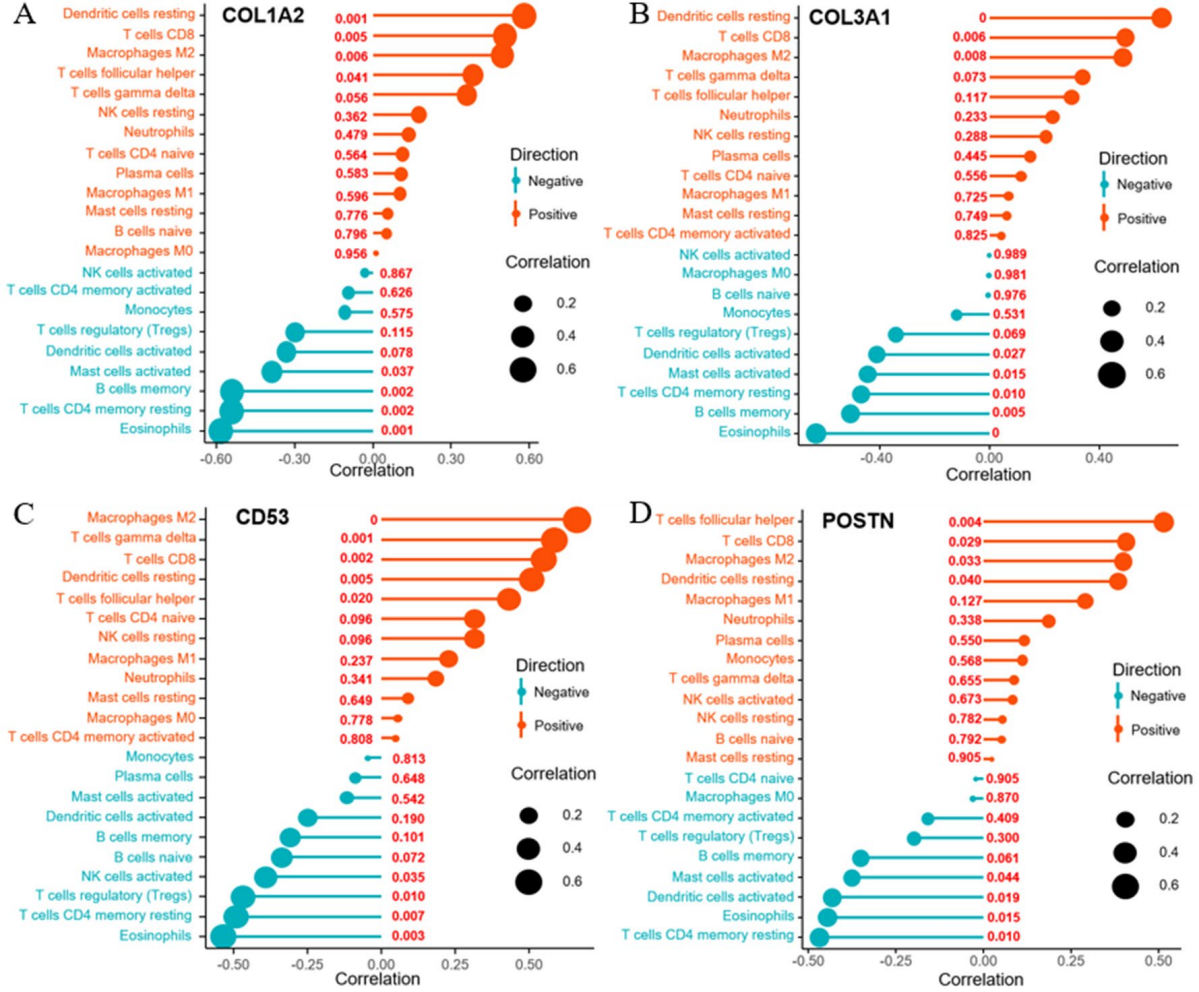
Correlation analysis of hub genes and infiltrated immune cells. The correlation analysis between infiltrated immune cells and four genes, including (**A**) *COL1A2*, (**B**) *COL3A1*, (**C**) *CD53*, and (**D**) *POSTN*. *P* < 0.05 represented statistical significance.

The relationship between the four hub genes and infiltrated immune cells was analyzed with the Spearman correlation analysis, and the results revealed that *COL1A2* was positively correlated with resting dendritic cells, CD8(+) T cells, M2 macrophages, and T follicular helper cells while negatively correlated with eosinophils, resting CD4(+) memory T cells, memory B cells, and activated mast cells (Fig. 6A). *COL3A1* was positively correlated with resting dendritic cells, CD8(+) T cells, and M2 macrophages, while negatively correlated with eosinophils, memory B cells, resting CD4(+) memory T cells, activated mast cells, and activated dendritic cells (Fig. 6B). *CD53* was positively correlated with M2 macrophages, gamma delta T cells, CD8(+) T cells, resting dendritic cells and T follicular helper cells while negatively correlated with eosinophils, resting CD4(+) memory T cells, regulatory T cells, and activated NK cells (Fig. 6C). *POSTN* was positively correlated with T follicular helper cells, CD8(+) T cells, M2 macrophages and resting dendritic cells while negatively correlated with resting CD4(+) memory T cells, eosinophils, activated dendritic cells, and activated mast cells (Fig. 6D). In summary, four hub genes were significantly associated with infiltrated immune cells, indicating all four hub genes may serve as potential immune-related genes in DCM.

### The expression and diagnosis value of potential immune-related genes

**Figure 7:**
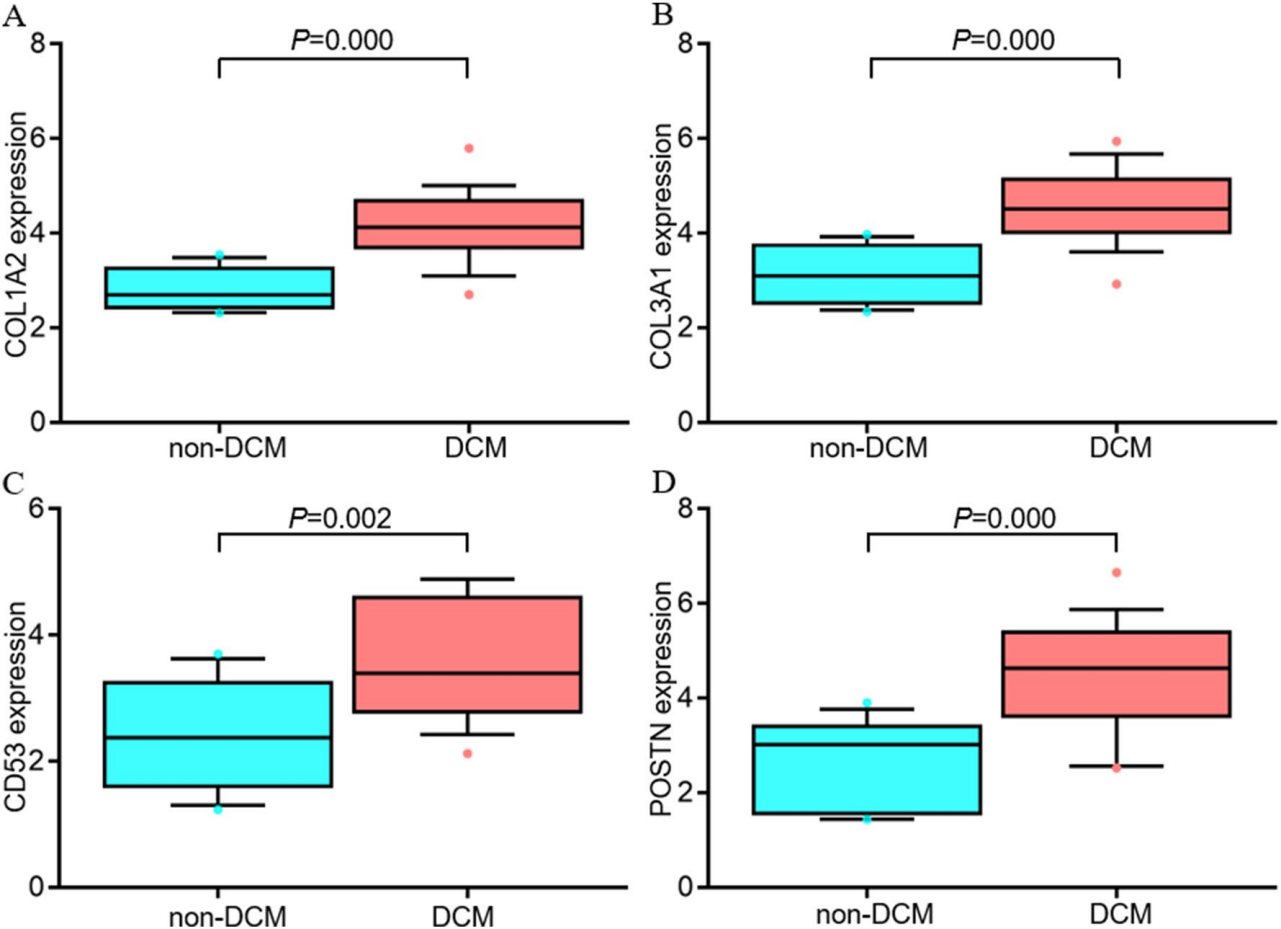
The expression levels of potential immune-related genes in DCM. (**A**) The expression level of *COL1A2*, (**B**) The expression level of *COL3A1*, (**C**) The expression level of *CD53*, and (**D**) The expression level of *POSTN*. DCM: dilated cardiomyopathy.

**Figure 8:**
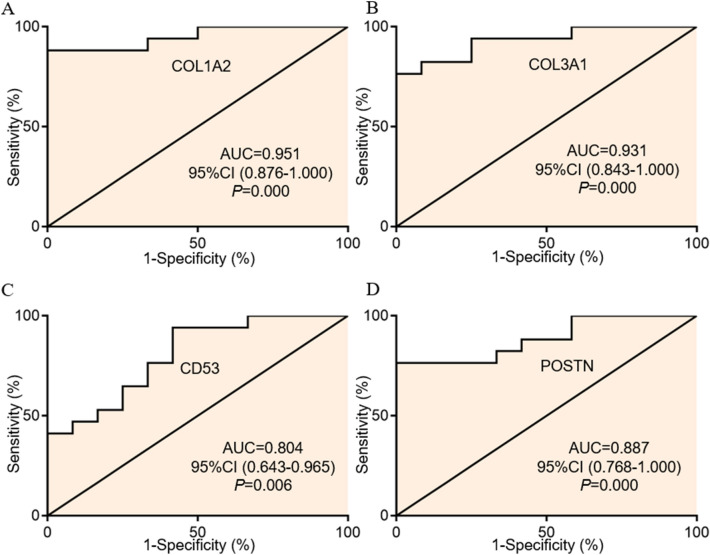
The diagnosis value for potential immune-related genes in DCM. (**A**) The ROC curves of *COL1A2*, (**B**) The ROC curves of *COL3A1*, (**C**) The ROC curves of *CD53*, and (**D**) The ROC curves of *POSTN*. DCM: dilated cardiomyopathy; ROC: receiver operating characteristic.

The general expressions of four potential immune-related genes (C*OL1A2*, *COL3A1*, *CD53*, and *POSTN*) were all significantly up-regulated in DCM patients (Fig. 7A–D). ROC curves were created and four potential immune-related genes showed AUCs were more than 0.7 (including 0.951 for *COL1A2*, 0.931 for *COL3A1*, 0.804 for *CD53*, and 0.887 for *POSTN*, respectively) (Fig. 8A–D), suggesting that the potential immune-related genes had a good discrimination for DCM from non-DCM.

### Identification of key immune-related genes

**Figure 9:**
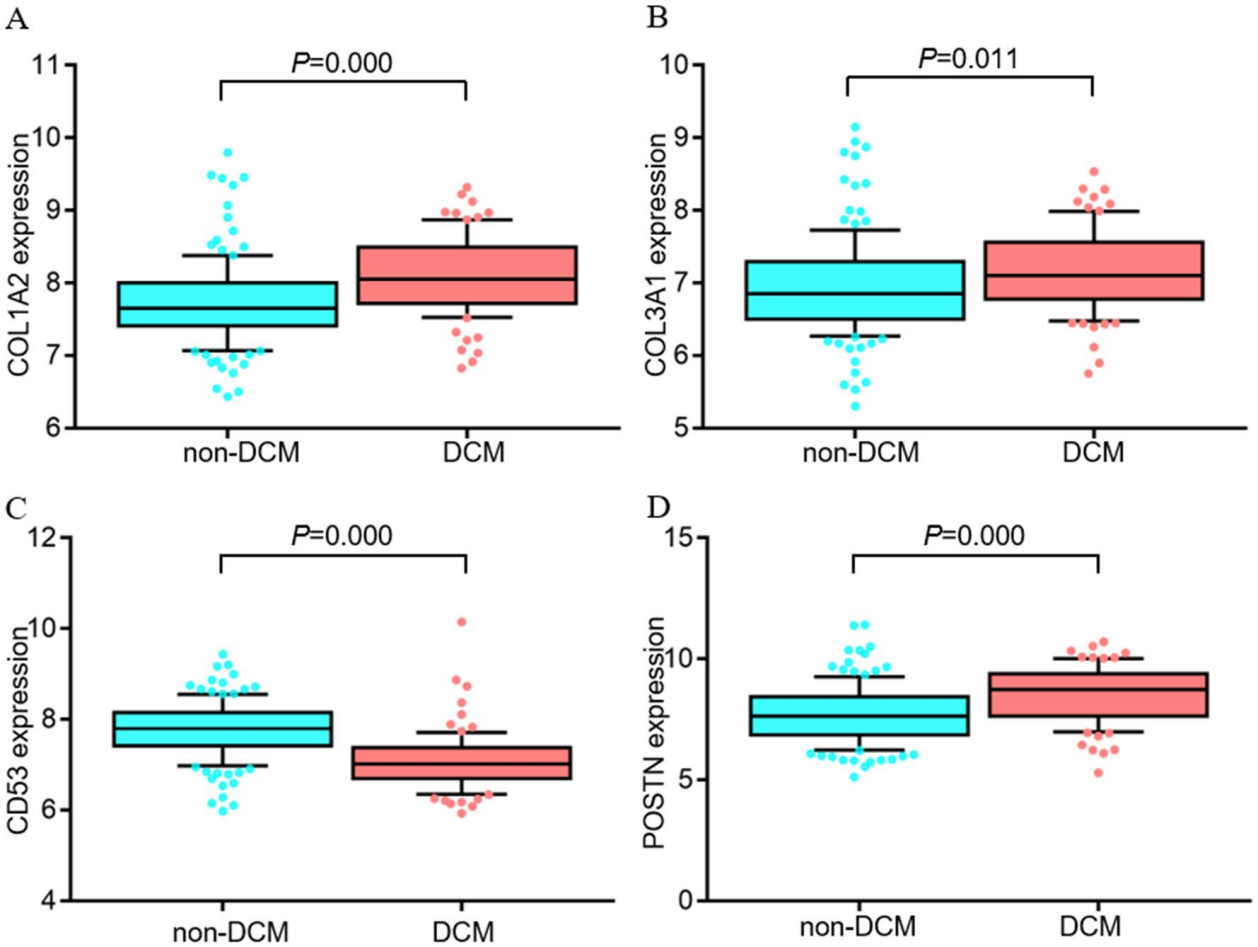
The expression levels of the four potential immune-related genes in validation set of GSE57338. (**A**) The expression level of *COL1A2*, (**B**) The expression level of *COL3A1*, (**C**) The expression level of *CD53*, and (**D**) the expression level of *POSTN*.

**Figure 10:**
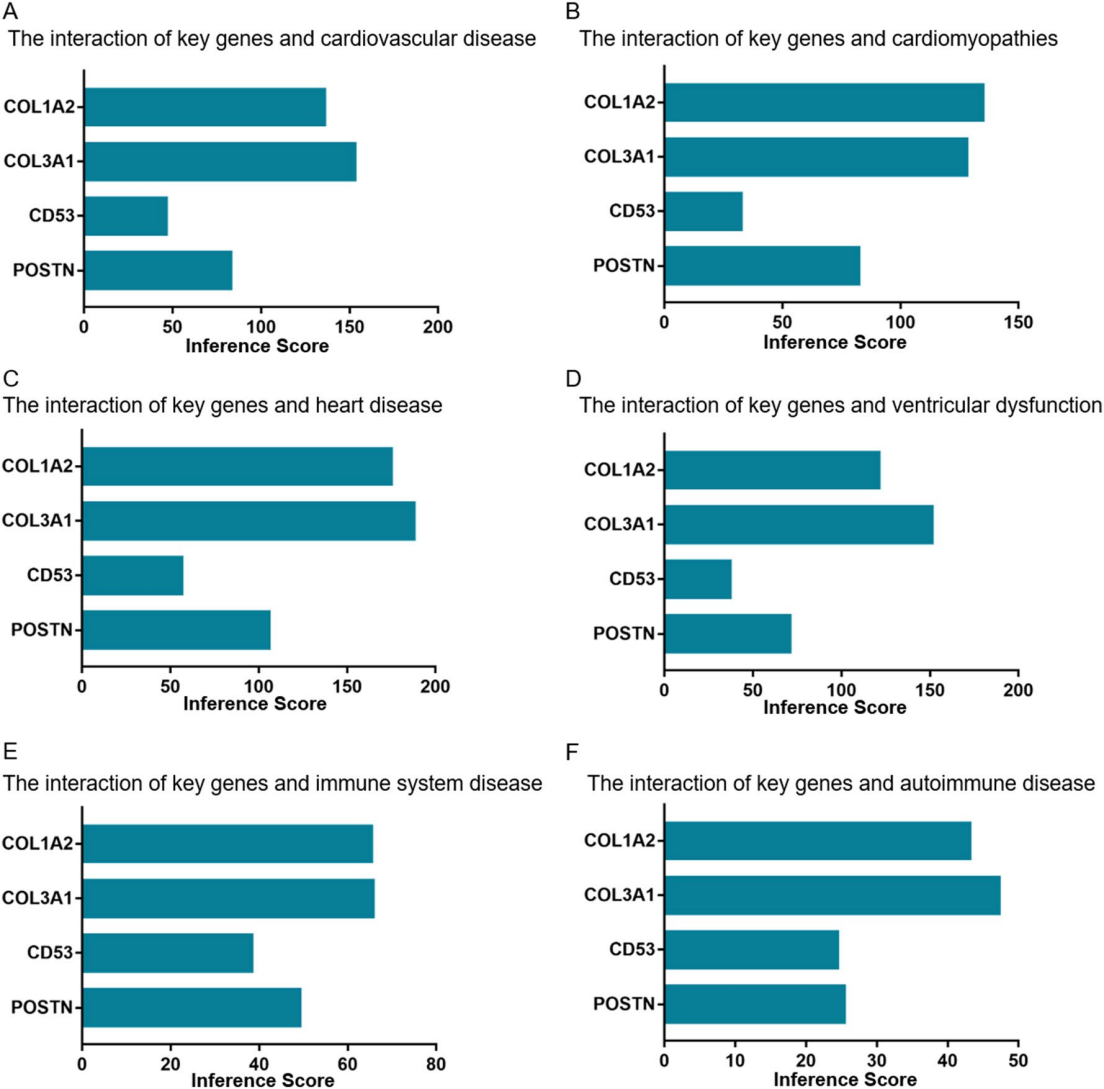
Relationship analysis of the four potential immune-related genes with cardiovascular and immune-related diseases in CTD. Four potential immune-related genes (*COL1A2*, *COL3A1*, *CD53*, and *POSTN*.) targeted multiple cardiovascular diseases and immune-related diseases, including (**A**) cardiovascular disease, (**B**) heart disease, (**C**) heart disease, (**D**) ventricular dysfunction, (**E**) immune system disease, (**F**) autoimmune disease, respectively. CTD: comparative toxicogenomics database.

The external dataset (GSE57338) was used for validation of the expression level of the four potential immune-related genes. The results showed that except for *CD53*, the expression levels of the remaining three genes were upregulated and were consistent with the integrated set (Fig. 9A–D). Moreover, the relationship analysis of the four potential immune-related genes with cardiovascular and immune-related diseases was performed using the online CTD database. The results suggested four genes targeted multiple cardiovascular diseases and immune-related diseases, including cardiovascular disease, cardiomyopathies, heart disease, ventricular dysfunction, immune system disease, and autoimmune disease) (Fig. 10A–F). Whereas the lowest interference score was displayed in the gene of *CD53* in the six diseases. Therefore, the three genes of *COL1A2*, *COL3A1*, and *POSTN* were identified as the key immune-related genes in DCM.

## Discussion

In this study, we screened a total of eighty DEGs between DCM patients and non-DCM patients using two DCM gene expression profiles from GSE3585 and GSE84796. We performed enrichment analysis to explore the potential mechanisms underlying DCM. We also conducted immune infiltration analysis for DCM patients. Then, PPI network was constructed and a total of four algorithms were used within Cytoscape to identify the hub genes based on PPI network. Correlation analysis between hub genes and infiltrated immune cells was performed to identify potential immune-related genes. The expression analysis and diagnosis value analysis of potential immune-related genes were performed. Finally, the expression analysis with validation set (GSE57338) and relationship analysis with CTD were performed to identify the key immune-related genes in DCM. The main findings are that: (1) Three key immune-related genes (C*OL1A2*, *COL3A1*, and *POSTN*) were identified between DCM patients and non-DCM patients; (2) The three up-regulated key genes might be implicated in the immune-related pathophysiological process in DCM. However, further studies are needed for illustrating the underlying mechanisms and providing potential therapeutic targets for DCM.

DCM, one of main types of cardiomyopathies, is characterized by the left ventricular or/and biventricular dilatation, ventricular wall thinner, and impaired myocardial systolic dysfunction, which ultimately leads to systolic heart failure. It has been reported that several cardiac risk factors and cardiovascular diseases may be involved in the DCM, including family history of cardiomyopathy, myocarditis, and toxic effects from illicit drugs, alcohol, or medications. Multiple physiopathology mechanisms had been reported to be implicated in the development of DCM, such as oxidative stress, abnormal immune response, inflammation, fibrosis and genetic changes. However, the key genes and precise molecular mechanisms underlying DCM, especially immune response abnormal, remain elusive. Interestingly, accumulated evidence had revealed that the abnormally innate and adaptive immune responses may play an essential role in the initiation and progression of DCM.

Preliminary studies had suggested that multiple immune cells, including cytotoxic CD8(+) T cells, B cells, and macrophages, were infiltrated in various types of DCM (such as Chronic Chagas cardiomyopathy and idiopathic dilated cardiomyopathies), in which T cells related differentiation might play an important role on the progression of abnormal immune and inflammatory response in DCM^6^. A recent study related to T cell subpopulations analysis, which based on the in silico approach and unsupervised machine learning methods, suggesting that a robust systemic inflammation and highly activated CD8(+) T cells were displayed in DCM patients^7^. Similarly, Fonseca et al.^8^ also revealed that the predominance and maintenance of CD8(+) T cell may be the key cell type in the tissue damage of Chagas disease induced DCM. T follicular helper cells, a specialized subset of CD4(+) T cells, have been demonstrated to provide help for B cells in the secondary lymphoid organs, which is significantly associated with multiple pathogenesis of diseases, including infectious diseases, allergies, and autoimmune diseases^9^. MHCII proteins, widely expressed in several immune cells (such as B cells, macrophages, and dendritic cells), could bind self or foreign proteins and participate the activation and regulation of adaptive immune response^10^. Increasing studies have indicated that MHC might sever as a marker for the abnormal immune response in multiple myocardial damage, including DCM^11,12^.

Reportedly, cardiac fibrosis, an essential feature in the progression of DCM, has been demonstrated to be associated with multiple immune cells, such as macrophages and T cells, which could directly or indirectly activate the cardiac fibroblasts^13^. Meanwhile, more and more studies had revealed that immune response abnormal could lead to inflammatory activation and fibrosis, especially in term of cardiovascular diseases^14–16^. A study comprising a total of 182 biopsy samples derived from consecutive DCM patients suggested that M2 macrophages infiltration was significantly related to the worse outcome (hazard ratio: 1.77, *P* < 0.05), and might be an independent determinant with collagen area fraction in DCM (*P* < 0.05)^17^. Also, nuclear receptors, a family of lipid- and hormone-activated transcription factors, including retinoic acid receptor, PPARγ, glucocorticoid receptor and, vitamin D receptor, were significantly associated with immunological recognition, immune regulation, and inflammatory immunity^18^.

Different from previous studies^19,20^, our enrichment analysis, in this study, for DEGs revealed that multiple enrichment items were significantly involved in immune response. Regulation of memory T cell differentiation, including regulation of T cell mediated cytotoxicity and immunological memory formation, has been mainly enriched in biological process. MHC class II protein complex and fibrillar collagen trimer were mainly enriched in cellular component. MHC class II receptor activity, nuclear receptor activity, and collagen binding were mainly enriched in molecular function. Importantly, enriched immune system processes for GO were mainly involved in the regulation of memory T cell differentiation and positive regulation of T cell mediated immunity, which further indicated that DEGs were associated with immunity. Cell adhesion and cell–cell interactions have been revealed as the crucial mediators for the activation of immune cells^21^. Interestingly, our KEGG analysis showed that the mainly enriched item was the cell adhesion molecules, which indicated an immunology biological pathway involved in DEGs. Moreover, immune infiltration analysis between DCM and non-DCM patients was performed. Consistent with previous studies^7,9,17^, we found that a higher proportion of CD8(+) T cell, T follicular helper cells, and M2 macrophages was shown in DCM patients, indicating that a potential abnormal immune regulation and response, as well as cardiac fibrosis, in DCM. In summary, DCM-related DEGs were involved in immune-related pathological process, highlighting a significant role of immunity in the development of DCM.

*COL1A2* (collagen type I alpha 2 chain) could encode the pro-alpha2 chain of type I collagen, which is widely distributed in most connective tissues, such as cornea, tendon, bone, and dermis. Preliminary studies showed that, except for accelerating the increase of extracellular collagen fibers, the up-regulated expression of *COL1A2* was significantly associated with immune cells infiltration in multiple diseases, including gastric cancer, radiation-induced lung injury, and Chagas disease cardiomyopathy^22–24^. Consistent with previous studies, our study showed was *COL1A2* also significantly up-regulated in DCM patients. Importantly, the results of correlation analysis of *COL1A2* and infiltrated immune cells revealed the up-regulated expression of *COL1A2* was positively correlated with immune activation related cells (e.g., CD8(+) T cells and M2 macrophages) and negatively correlated with immune homeostasis related cells (e.g., eosinophils, memory B cells, and resting CD4(+) memory T cells). These results indicated that the up-regulated *COL1A2* expression may promote the activation of immune system and aggravate the immune-dependent injury, thus facilitating the development of DCM.

In DCM, the cardiac fibrosis, an important pathological characteristic, could be activated by multiple humoral and cellular factors, leading to the increased cardiac rigidity, decreased myocardial performance, and enhanced sudden death risk. Accumulated studies had revealed various immune cells (e.g., macrophages, CD8(+) T cells, and activated B cells) were involved in the development of cardiac fibrosis via directly activating cardiac fibroblasts or/and indirectly releasing pro-fibrotic cytokine^13,25^. *COL3A1*, also named collagen type III alpha 1 chain, plays a significant role in encoding fibrillar collagen, contributing to the progression of fibrosis in extensible connective tissues such as lung, skin and the vascular system. Consistent with our study, multiple researches had indicated an elevated expression of *COL3A1* in DCM patients compared with non-DCM patients. Guo et al.^26^ established coxsackie virus B3 induced DCM mice model, and the results showed the up-regulated *COL3A1* expression companied with immune cells infiltration (e.g., T helper cells) and myocardial fibrosis. Similarly, our results showed the elevated *COL3A1* expression was positively correlated with CD8(+) T cells, M2 macrophages, and T follicular helper cells, while also negatively correlated with eosinophils, resting CD4(+) memory T cells, and memory B cells, which suggested *COL3A1* may sever as a key immune-related gene playing a central role of immune mechanism in the progression of DCM.

*POSTN* is also named periostin, and encodes a secreted extracellular matrix protein, playing a significant role in tissue development and regeneration, including wound healing, and ventricular remodeling post myocardial infarction. An increasing number of researches revealed *POSTN* was significantly associated with immune response abnormality, inflammation, and fibrosis^27–29^. Similarly, our study showed the elevated *POSTN* expression was positively correlated with T follicular helper cells, CD8(+) T cells, and M2 macrophages while negatively correlated with resting CD4(+) memory T cells and eosinophils. These results suggested *POSTN* may be a key immune-related gene in DCM.

## Limitations

Several limitations in this study should be acknowledged. First, a significant limitation is that all the findings are just microarray analysis that based on the DCM gene expression value and lack of confirmatory experiments. Whereas, it would be reasonable to conduct a comprehensive analysis using the economical and effective resource for screening the potential mechanisms of DCM. Therefore, our study is just descriptive analysis, and lack of mechanisms exploration, and further experiments are needed to verify our results. Second, the data set GSE84796 belongs to the DCM gene expression profile with relatively clear etiology (infectious disease), which might lead to potential bias in our analysis. Meanwhile, the DCM data with Chagas disease (GSE84796) might be partly responsible for screening a strong inflammatory and cardiac remodeling pathway and biological process. Whereas, two gene expression profiles (GSE3585 and GSE84796) with combined analysis might facilitate to screen the immune-related, typical and mutual genes, as well as physiopathology mechanisms for the initiation and progress of DCM. Third, though a total of two datasets (GSE3585 and GSE84796) were analyzed, the input data might be still insufficient to exactly screen and identify the hub genes for DCM. However, a comprehensive and superior method was performed to screen immune-related genes (such as application of multiple algorithms within Cytoscape, immune infiltration, as well as correlation analysis between hub genes and infiltrated immune cells), and another gene expression profile (GSE57338, including a total of 218 samples) was used for validation the expression level of potential immune-related genes for DCM. All these facilitated improve the accuracy and reliability of our results to a great extent. Finally, the DCM samples of GSE3585 and GSE84796 were derived from subendocardial left ventricular tissue, and left ventricular free wall tissue, respectively. Considering the heterogeneity of the DCM samples^30^, our results might have a selection bias. Meanwhile, multiple details (e.g. age, gender, ethnicity, body mass index, and comorbidities) for GSE3585 and GSE84796 are not available from GEO platform, which also increased some potential heterogeneity due to clinical confounding factors.

## Supplementary Information

Supplementary Information 1.

Supplementary Information 2.

Supplementary Information 3.

Supplementary Information 4.

Supplementary Information 5.

Supplementary Information 6.

Supplementary Information 7.

Supplementary Information 8.

Supplementary Information 9.

Supplementary Information 10.

Supplementary Information 11.

Supplementary Figure 1.

## Data Availability

The datasets generated and analyzed during the current study are available in the public Gene Expression Omnibus database (https://www.ncbi.nlm.nih.gov/geo/query/acc.cgi?acc=GSE3585; https://www.ncbi.nlm.nih.gov/geo/query/acc.cgi?acc=GSE84796; https://www.ncbi.nlm.nih.gov/geo/query/acc.cgi?acc=GSE57338). The data are available from the corresponding author (Ru-Xing Wang) on reasonable request.
