# Publisher Correction: Identification of key immune-related genes in dilated cardiomyopathy using bioinformatics analysis

**DOI:** 10.1038/s41598-023-30137-6

**Published:** 2023-02-22

**Authors:** Feng Li, Tong‑Yue Du, Li‑Da Wu, Lei Zhang, Huan‑Huan Liu, Zhen‑Ye Zhang, Jie Zhang, Zhi‑Yuan Zhang, Ling‑Ling Qian, Ru‑Xing Wang, Jian‑Feng Hao

**Affiliations:** 1 Department of Cardiology, Wuxi People’s Hospital Affiliated to Nanjing Medical University, No. 299, Qingyang Road, Wuxi, 214023 China; 2 Department of Critical Care Medicine, The Second Hospital of Nanjing, Nanjing University of Chinese Medicine, Nanjing, 210003 China; 3 Wuxi School of Medicine, Jiangnan University, Wuxi, 214122 China; 4 Department of Cardiopulmonary Rehabilitation, Wuxi Tongren Rehabilitation Hospital Affiliated to Nanjing Medical University, Wuxi, 214122 China

Correction to: *Scientific Reports* 10.1038/s41598-022-26277-w, published online 01 February 2023

The original version of this Article contained an error in the order of the author names, which was incorrectly given as Feng Li, Tong-Yue Du, Li-Da Wu, Lei Zhang, Huan-Huan Liu, Zhen-Ye Zhang, Jie Zhang, Zhi-Yuan Zhang, Ling-Ling Qian, Jian-Feng Hao & Ru-Xing Wang.

The original Article has been corrected.

