# Supplementary material for: Identification of key immune-related genes in dilated cardiomyopathy using bioinformatics analysis
Source: Sci Rep. 2023 Feb 1;13:1820. doi: 10.1038/s41598-022-26277-w (PMC9892583; doi:10.1038/s41598-022-26277-w)
Supplement: Supplementary file 12 — Supplementary Figure 1. [file 41598_2022_26277_MOESM12_ESM.docx]

**Supplementary Figure**


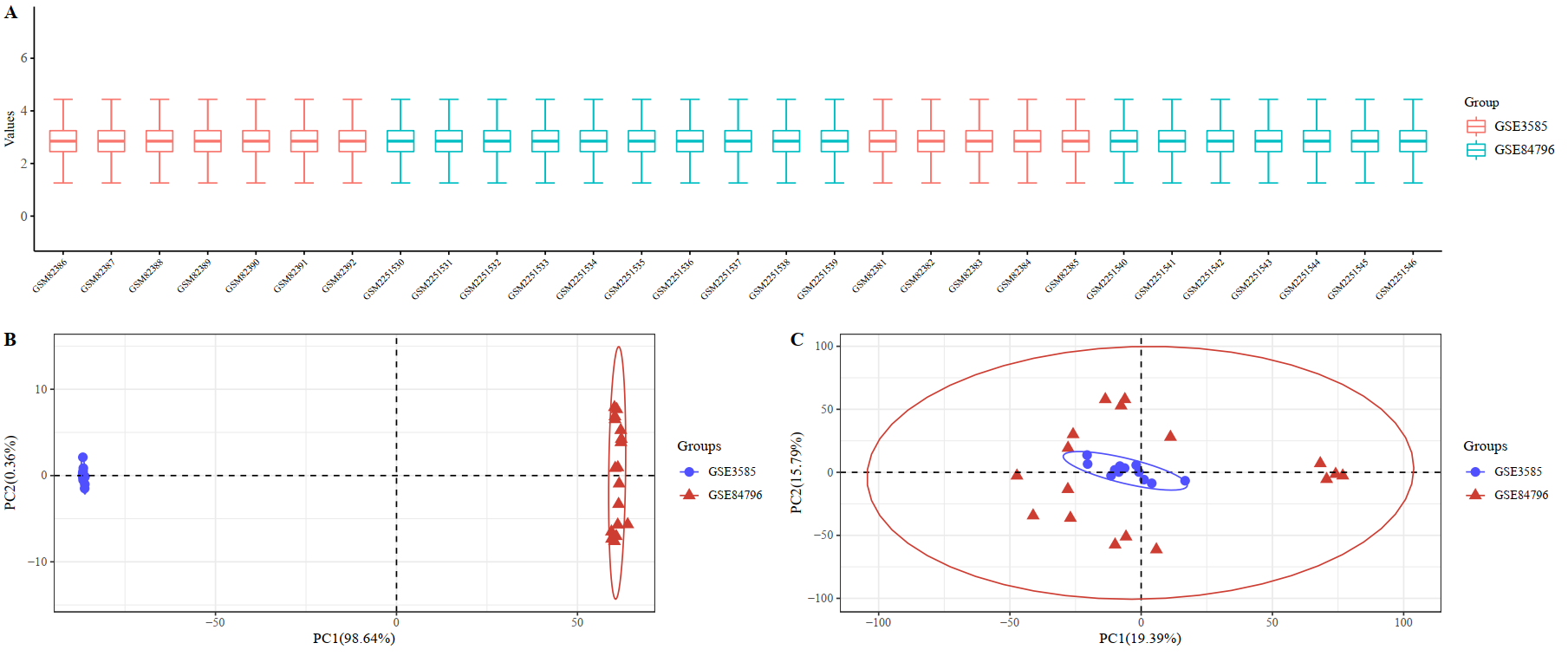


**Supplementary Figure 1.** Data procession for the DCM gene expression profiles of GSE3585 and GSE84796. (A) The result of data standardization for the two DCM gene expression profiles; (B-C) The PCA results between gene expression profiles before and after batch removal.
